# Building the capacity of policy-makers and planners to strengthen mental health systems in low- and middle-income countries: a systematic review

**DOI:** 10.1186/s12913-016-1853-0

**Published:** 2016-10-21

**Authors:** Roxanne Keynejad, Maya Semrau, Mark Toynbee, Sara Evans-Lacko, Crick Lund, Oye Gureje, Sheila Ndyanabangi, Emilie Courtin, Jibril O. Abdulmalik, Atalay Alem, Abebaw Fekadu, Graham Thornicroft, Charlotte Hanlon

**Affiliations:** 1 South London and Maudsley NHS Foundation Trust, Maudsley Hospital, Denmark Hill, London, SE5 8AZ UK; 2 Centre for Global Mental Health, King’s College London, Institute of Psychiatry, Psychology and Neuroscience, 16 De Crespigny Park, London, SE5 8AF UK; 3 Department of Psychiatry, University of Oxford, Warneford Hospital, Warneford Lane, Oxford, OX3 7JX UK; 4 Health Service and Population Research Department, Institute of Psychiatry, Psychology & Neuroscience, King’s College London, De Crespigny Park, London, UK; 5 PSSRU, London School of Economics and Political Science, Houghton Street, London, WC2A 2AE UK; 6 Department of Psychiatry and Mental Health, Alan J Flisher Centre for Public Mental Health, University of Cape Town, 46 Sawkins Road, Rondebosch, 7700 South Africa; 7 Department of Psychiatry, College of Medicine, University of Ibadan, Ibadan, Nigeria; 8 Ministry of Health, PO Box 7272, Kampala, Uganda; 9 London School of Economics and Political Science, Social Policy Department – LSE Health and Social Care, Houghton Street, London, WC2A 2AE UK; 10 College of Health Sciences, School of Medicine, Department of Psychiatry, Sixth Floor, College of Health Sciences Building, Addis Ababa University, Tikur Anbessa Hospital, PO 9086, Addis Ababa, Ethiopia; 11 College of Health Sciences, School of Medicine, Department of Psychiatry, Addis Ababa University, PO 9086, Addis Ababa, Ethiopia; 12 Department of Psychological Medicine, Institute of Psychiatry, Psychology and Neuroscience, King’s College London, Centre for Affective Disorders, London, UK; 13 Centre for Global Mental Health and Centre for Implementation Science, King’s College London, Institute of Psychiatry, Psychology and Neuroscience, 16 De Crespigny Park, London, SE5 8AF UK; 14 Addis Ababa University, College of Health Sciences, School of Medicine, Department of Psychiatry & Centre for Global Mental Health, King’s College London, Institute of Psychiatry, Psychology and Neuroscience, 16 De Crespigny Park, London, SE5 8AF UK

**Keywords:** Mental health, Capacity-building, Policy-makers, Health system strengthening, LMICs, Systematic review, Psychiatry, Global mental health

## Abstract

**Background:**

Little is known about the interventions required to build the capacity of mental health policy-makers and planners in low- and middle-income countries (LMICs). We conducted a systematic review with the primary aim of identifying and synthesizing the evidence base for building the capacity of policy-makers and planners to strengthen mental health systems in LMICs.

**Methods:**

We searched MEDLINE, Embase, PsycINFO, Web of Knowledge, Web of Science, Scopus, CINAHL, LILACS, ScieELO, Google Scholar and Cochrane databases for studies reporting evidence, experience or evaluation of capacity-building of policy-makers, service planners or managers in mental health system strengthening in LMICs. Reports in English, Spanish, Portuguese, French or German were included. Additional papers were identified by hand-searching references and contacting experts and key informants. Database searches yielded 2922 abstracts and 28 additional papers were identified. Following screening, 409 full papers were reviewed, of which 14 fulfilled inclusion criteria for the review. Data were extracted from all included papers and synthesized into a narrative review.

**Results:**

Only a small number of mental health system-related capacity-building interventions for policy-makers and planners in LMICs were described. Most models of capacity-building combined brief training with longer term mentorship, dialogue and/or the establishment of networks of support. However, rigorous research and evaluation methods were largely absent, with studies being of low quality, limiting the potential to separate mental health system strengthening outcomes from the effects of associated contextual factors.

**Conclusions:**

This review demonstrates the need for partnership approaches to building the capacity of mental health policy-makers and planners in LMICs, assessed rigorously against pre-specified conceptual frameworks and hypotheses, utilising longitudinal evaluation and mixed quantitative and qualitative approaches.

**Electronic supplementary material:**

The online version of this article (doi:10.1186/s12913-016-1853-0) contains supplementary material, which is available to authorized users.

## Background

The global burden of mental, neurological and substance use (MNS) disorders is high, resulting from chronic disability combined with premature mortality [1, 2]. Untreated MNS disorders also impact negatively on global health priorities [3], and may be associated with human rights abuses [4]. The majority of people with MNS disorders in LMICs are unable to access effective mental health care, with the treatment gap higher than 90 % in some low-income countries [5].

Health system constraints are potent threats to the scale-up of evidence-based mental health care for people affected by MNS disorders in LMICs [6]. Policy-makers and planners play a critical role in the successful strengthening of mental health systems, but despite historical attempts at capacity-building [7], may not be appropriately equipped for the task. The limited evidence and evaluation of capacity-building interventions for policy-makers in LMICs has been recognized in physical health care [8] and is likely to be even more significant in mental health care. In a mixed-methods report of implementing a national mental health policy in South Africa, barriers included the relatively low priority given to mental health care by planners, provincial bureaucracy around service coordination, insufficient staff for policy-making and service planning, and disinclination by some local authorities to lead mental health policy implementation [9].

In a qualitative study involving national and regional stakeholders in Ghana, South Africa, Uganda and Zambia, low perceived legitimacy of the problem of scaling up mental health services and inadequate government support were identified as factors perpetuating the low priority accorded to mental health care [10]. A qualitative survey of leaders and specialists in international mental health specifically identified the need for a broader and more holistic view of mental health care, adopting a more public health-level perspective among mental health policy-makers [11]. In particular, the lack of training and experience of clinicians to fulfil leadership roles was emphasized as a barrier to high quality mental health policy-making.

There is international consensus on the need for mental health system strengthening and for a specific focus on building the capacity of key stakeholders, including policy-makers and planners, and service users [12]. ‘Systems thinking’ casts health services as a type of complex adaptive system, characterized by large numbers of individuals occupying a range of roles, acting in the context of and adapting to constant internal and external changes [13]. It advocates a ‘systems perspective’ which unifies health care with education, research and policy-making, through “collaboration across disciplines, sectors and organizations; ongoing, iterative learning; and transformational leadership” [14]. This approach in part represents a reaction to the former emphasis on targeted, often disease-specific health investments, which have not demonstrated benefits for the wider health system [15]. However, the specific means by which the capacity of mental health policy-makers and planners should be built, to facilitate such systems-wide scaling up, are less well-documented. A recent systematic review of the involvement of mental health service users and their caregivers in mental health system strengthening, identified a lack of high quality research [16].

Given the widespread support for building the capacity of policy-makers and planners in LMICs to achieve mental health system strengthening, this systematic review aimed to identify the best evidence and experience for specific capacity-building intervention models. A secondary aim was to identify methods of evaluating models of capacity-building for mental health policy-makers and planners in LMICs.

## Methods

This systematic review formed part of the ‘Emerging mental health systems in LMICs’ (Emerald) program, which focuses on the health system inputs, processes and outputs required for mental health service scale-up in LMICs [17]. A systematic investigation of existing evidence-based strategies was an important precursor of the Emerald project, since one of its aims is to build the capacity of policy-makers and planners to strengthen the mental health system. This work was registered on the PROSPERO international prospective register of systematic reviews (Registration Number: CRD42016032798).

### Eligibility criteria

In view of the anticipated scarcity and heterogeneity of relevant literature, the criteria used for selection of studies into this review were intentionally broad and inclusive. The review set out to include any type of study design, review or report on the evidence, experience or evaluation of capacity-building of policy-makers, service planners or managers in mental health system strengthening in LMICs. Studies were included whenever the recipients of system-focused capacity-building were either currently involved in policy-making, planning or co-ordination of services, or when recipients had the potential to take on such a role, regardless of their professional background. Mental health systems were interpreted to include services addressing the priority MNS disorders outlined in the World Health Organization’s mental health Gap Action Program [18]. Papers written in the following languages were included: English, Spanish, Portuguese, French or German. Studies were excluded where system level interventions to support implementation and expansion of mental health care were implemented by external agencies and did not report specifically on the building of local capacity. A common reason for exclusion was that studies described mental health training of clinical staff without discussing training needs of mental health policy-makers, or commented on the need for capacity-building without presenting any interventions to build policy-makers’ capacity. Studies that reported solely from high-income countries were excluded.

### Search strategy

The following databases were searched on 12 November 2013: MEDLINE (from 1946 until December 2013), Embase (1974 to December 2013), PsycINFO (1806 to December 2013), Web of Knowledge, Web of Science, CINAHL, LILACS, ScieELO, Google Scholar and Cochrane and on 27 November 2013, Scopus (all from the start date of the database to November 2013). The search was updated on 25 November 2015 to look for any papers published in the intervening period of two years. Details of the search strategy used are given in Additional file 1. In addition to database searches, the reference lists of included papers were hand-searched for relevant studies and experts in the field were contacted to identify any further studies. Grey literature including reports and web-based resources were identified via the Google Scholar search and experts in the field.

### Study selection and data extraction

**Fig. 1:**
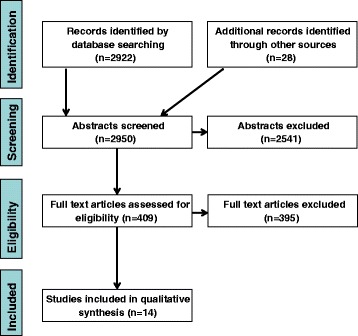
PRISMA flow diagram for selection of peer-reviewed articles

Figure 1 shows the flow of papers from identification to selection and data extraction. The titles and abstracts of all 2950 papers identified in the search were assessed by two independent reviewers. This number comprises 2040 papers identified in the first database search up to November 2013, a further 882 papers identified in the second database search up to November 2015 and 28 papers identified by contacting experts or key informants, hand-searching references, and searching the internet. In the first search, the 1704 papers excluded at the stage of abstract review were either not eligible for inclusion due to their subject matter (1581), because they were duplicates (66), based in high income countries (51) or due to language (6). In the second search, 837 papers were excluded at the stage of abstract review because of ineligible subject matter (827), based in high income countries (8) or due to language (2). Papers were considered to pertain to LMICs if the work described took place in a year in which the country was classified by the World Bank [19] as a low or middle-income country.

This process yielded a total of 409 papers which were reviewed as full text articles. For the screening of full text papers identified by the database search, all articles were reviewed independently by at least two reviewers (RK, SE-L, JA or MT). Additional papers identified via experts and informants and hand-searching references and the internet were screened independently by SE-L, JA, MT or RK. Where there was disagreement between independent reviewers of full text articles regarding inclusion, the paper was reviewed by two senior independent reviewers (CH and MS), who made a final decision. In several papers, the language used to describe participant groups and interventions was vague, creating ambiguities about whether the work constituted clinical training alone or clinical training combined with capacity-building. In such cases, papers were reviewed again and where necessary, input from a third independent reviewer was obtained. MS reviewed 7 full texts where there was disagreement, leading to inclusion of 4 studies. CH reviewed 5 full texts where there was disagreement and included 3 studies. This was sufficient in all cases and contacting the authors of such papers was not required.

Of the 409 full text papers screened, 395 were excluded due to not addressing the focus of this review: capacity-building interventions for mental health policy-makers and planners.

Data from included papers were extracted by independent reviewers (RK, MT and JA) and inputted into a data extraction form (Additional file 2). The data extracted included: authorship (noting whether or not this included authors from LMICs), type of publication, countries involved, study design, capacity-building participants, sample size, nature of capacity-building and any guiding framework, type of evaluation, type of evaluation data and a summary of findings.

#### Quality evaluation

Given the mixed methods employed by included studies, their quality was evaluated using two methods: one to assess quantitative aspects (Additional file 3) and one to assess qualitative aspects (Additional file 4). Included papers were assessed by MT and RK using the Effective Public Health Practice Project (EPHPP) Quality Assessment Tool for Quantitative Studies [20], which rates studies from 1 (‘strong’) to 3 (‘weak’) for selection bias, study design, confounders, blinding, data collection method, withdrawals and dropouts. These scores are used to obtain a global rating of strong (no ‘weak’ ratings), moderate (one ‘weak’ rating) or weak (two or more ‘weak’ ratings). Included papers were also evaluated by MT or RK against 12 criteria for high quality qualitative research, derived from the literature and previously employed to distinguish papers of low, medium and high quality [21, 22]. The specific criteria are included in a footnote to Additional file 4 and pertain to quality of reporting, use of strategies to increase reliability, validity and rigor. Studies were assessed as of low quality if they met seven criteria or fewer, medium quality if they met between seven and nine criteria and high quality if they met ten or more criteria.

## Results

Following the process of systematic searching, 14 papers fulfilled the inclusion criteria and were included in this review. The data extracted from included papers were not amenable to statistical synthesis through meta-analysis, being heterogeneous and largely non-numerical. The study findings were therefore reviewed in a narrative manner instead.

### Included papers: capacity-building interventions for mental health policy-makers and planners

The 14 papers included in the review described nine capacity-building interventions. One intervention aimed to equip policy-makers and planners with “knowledge, skills and attitudes required to make the paradigm shift and the translation into the effective provision of health care” for people with substance use disorders [23]. Another implemented a range of interventions to scale up a harm reduction program for people who inject opioids [24]. Three interventions of the nine were orientated towards developing skills in planning and co-ordination of mental health care [25–30] and a fourth did so with a specific focus on de-institutionalization of children’s services [31]. Two programs focused specifically on enhancing mental health leadership [32–35] and a further intervention aimed to build regional capacity to prevent and control mental illness and other non-communicable diseases (NCDs) across 24 countries [36].

Each capacity-building intervention combined short training courses or workshops with a system of continued mentorship or dialogue. In most cases the interventions were not specific to policy-makers and planners but to any person involved in mental health service co-ordination, planning or development, including mental health professionals. The nine capacity-building interventions described by the papers included in this review are summarized in Table 1.

**Table 1:** Summary of capacity-building interventions

Capacity-building intervention	Summary
Capacity-building for substance misuse system change in Russia [22]	As part of a larger program of mental health service enhancement, a project was implemented in St Petersburg, to support a shift from a universally medical model of drug and alcohol services to one which incorporates psychosocial aspects of care. Through a program of exchange between Russia and England, staff from the University of Portsmouth and St Petersburg paid learning visits to each country’s services and shadowed peers, supplemented by seminars and workshops on different therapies and clinical skills, such as patient assessment and motivational interviewing, followed by supervision sessions. Training participants included staff with responsibilities for policy-making and implementation.
Scaling up harm reduction for opioid injecting in India [23]	Project ORCHID (organized response for comprehensive HIV interventions in selected high-prevalence districts) aimed to establish and scale-up a harm reduction package for people injection opioid drugs in two states of north-eastern India, through capacity-building of community participation and a network of 24 local non-government organizations (NGOs). They created a program management team comprising injecting opioid users, which gradually took responsibility for supporting local advocacy work. Through its emphasis on project management and support, ORCHID fostered data-driven decision-making, development of guidelines and training manuals to standardize care and widespread, robust monitoring procedures. NGO-led strategies such as task-shifting, harm reduction methods, opioid agonist treatment, mobile outreach and peer education were introduced, which later influenced national policy.
Mental health system strengthening in Russia [24]	In a partnership between the Institute of Psychiatry, London and the Russian Federal Government, WHO, local municipalities and universities led the implementation of a multifaceted intervention in urban, semi-urban and rural areas of Sverdlovsk oblast. Following a context-specific literature review and situational assessment, interventions implemented over two years incorporated local and government-level policy dialogues, creation of Inter-sectoral Steering Committees (ISCs) to facilitate patients’ access to health care and other services, mental health training for social workers and general medical doctors. In addition, training and technical support were provided to build the capacity of non-government organizations in the fields of advocacy, service provision, and management.
Mental health system strengthening in Kenya and Tanzania [25–28]	Two diverse mental health system strengthening programs were implemented in Kenya and Tanzania, each comprising initial country-level situation analysis, local and national multidisciplinary policy dialogue, a system to coordinate mental health services and mental health training and supervision, establishment of clinical guidelines, inter-sectoral partnerships from local to national levels, awareness-raising activities and work to incorporate mental health into general health systems. In Kenya, alongside training of primary care workers, capacity-building courses were delivered to provincial, district, and sub-district general hospitals, to promote the incorporation of mental health into local annual operational plans (which feed into the national annual operational plan), and to foster supervision and coordination of primary mental health care by secondary care psychiatric and public health nurses.
Mental health capacity-building in Nicaragua [29]	In a four year collaboration between the National Autonomous University of Nicaragua in León and the Centre for Addiction and Mental Health in Canada, a needs assessment was conducted for mental health capacity-building. Two international workshops on mental health and addictions focusing on primary care were delivered to clinical staff, academics, and NGO members, after which diploma and masters-level courses were developed, to enhance inter-professional leadership, knowledge exchange, networking, and education.
De-institutionalization of children’s services in Russia [30]	From 2009 to 2012, a two-tier educational program was implemented in the Nizhny Novgorod region of Russia, to establish a model of children’s community services to reduce institutionalization. In the first tier, five week-long seminars delivered a course introducing principles of managing children’s community services with a focus on early intervention and family support to experienced clinical staff motivated to take on roles in service planning. In the second tier, a mentorship and training program was delivered over two years to staff at eight pilot sites and additional staff from five ‘participant sites’, covering the knowledge and skills to lead family support and early intervention programs in children’s services. During this period, four week-long supervisory seminars created opportunities for staff to assess progress, receive service user feedback, and discuss challenges faced.
The International Mental Health Leadership Program (iMHLP) [31–33]	Founded in 2001, the capacity-building intervention of iMHLP is embedded within a broader program of mental health system strengthening housed at the University of Melbourne’s Centre for International Mental Health. A collaboration with the University’s Department of Global Health and Social Medicine and Harvard Medical School, iMHLP’s goal is “to contribute to the development of effective mental health systems by providing training and mentoring in leadership”. The program focuses on training future leaders of mental health systems in LMICs. It comprises a four week intensive workshop, complemented with subsequent provision of mentoring and supervision to participants at home. The program encompasses skills in leadership, policy, mental health budgeting, community service design, human resources, advocacy, human rights, and research aspects. Course attendees undertake a research or service development project, with support from staff at the University of Melbourne and seniors in their home country. Examples of previous participants include key stakeholders in the development of community mental health systems in both Sri Lanka and the Indonesian province of Aceh, whose health and social care services were severely affected by the 2004 Indian Ocean earthquake and tsunami.
The mental health Leadership and Advocacy Program (mhLAP) [34]	mhLAP represents a collaboration between the Department of Psychiatry, University of Ibadan, Nigeria, the international development organization, CBM International, and the Center for International Health, University of Melbourne. Commencing in 2010, the program’s objectives are “capacity-building for mental health leadership and advocacy” and “development of stakeholder groups with the ability to identify and pursue country-specific mental health service development needs and targets”. The program comprises an annual two week interactive training course, whose curriculum was influenced by that of the iMHLAP, including research developments in public health and health system development, and stakeholder needs in LMICs. The course covers the “burden of mental, neurological, and substance use disorders, organization of mental health services, evaluation of services, mental health financing, mental health policy and legislation, social determinants of mental health, principles and practice of health promotion, the art of communication, stigma of mental illness, and mental health system reform and strengthening. The course also aims to inform the trainees about the strong reciprocal links that exist, at both personal and national levels, between poverty, social determinants, and mental health.” In addition, a country facilitator is appointed in each of the participating countries in West Africa. Their role includes performing a baseline assessment of local mental health services, to inform later program evaluations. The role additionally entails building connections between mental health stakeholders and supporting activities identified as their priorities. This proceeds through creation of a National Stakeholders Council (NSC) or support for existing local networks. An annual three day workshop is held in Nigeria for NSC representatives, to facilitate regular monitoring and review, and sharing of experiences from each country’s activities.
The Consortium for NCD prevention and control in sub-Saharan African (CNCD-Africa) [35]	At an international meeting of stakeholders, including representatives from the United States Centers for Disease Control and Prevention (CDC), the Department of Health of England, the International Union for Health Promotion and Education, and World Health Organization, CNCD-Africa was established. Its objectives were to provide a framework for dialogue about NCDs in sub-Saharan Africa, to bring together regional activities in NCD prevention and control, to develop policies, standards, guidelines, and protocols, and mobilize regional resources, to foster networking, partnership, and evaluation of NCD interventions, and identify causative factors and communicate results worldwide. Mental health was included among NCDs and regional capacity-building took place through convening of symposia and meetings, representation of CNCD-Africa at external meetings, creation of two open-access documents on NCD policies in the region, local and international promotion, and awareness raising and networking through social media.

### Methods of evaluating capacity-building interventions for mental health policy-makers and planners

The strength of evidence to support any particular capacity-building approach for policy-makers and planners was low. One study used a conceptual framework for evaluation, which included the collection of primary evaluation data [23]. In that case, external and internal evaluation processes were employed to collect complementary data on the success of the program. The external evaluator collected data from documentary analysis of subsequent funding applications, project manager reports, telephone and face-to-face interviews with UK partners and Russian participants. This was combined with an ‘insider’ evaluation influenced by ‘utilization-focused’ approaches [37]. The authors applied a systematic framework used in previous substance misuse research [38] because it was considered to account for the complex nature of cross-cultural education and training of health care staff. The mental health leadership and advocacy (mhLAP) intervention embraced a systematic approach to evaluation, with consideration of numerical process indicators, such as numbers of policy-makers and planners participating in the national stakeholder council, output indicators, such as numbers of advocacy activities by type and outcome indicators, such as preparation of a country-specific situational analysis report [35].

For the other capacity-building interventions, a descriptive account of how the mental health system had been strengthened was given, but with no clear methodology to guide the evaluation. In none of the reports did the evaluation methodology attempt to isolate the impact of the capacity-building intervention from other contextual factors that might also have led to the reported outcomes. For example, when multi-faceted interventions were implemented which targeted both clinical service delivery and capacity-building of policy-makers and planners, efforts were not made to independently assess the impact of capacity-building activities, in isolation.

### Impact of capacity-building interventions for mental health policy-makers and planners

The 14 papers included in this narrative review presented evidence of success, including process indicators (e.g. number of people trained within the program), output indicators (e.g. participation of trained people in mental health system strengthening activities) and outcome activities (e.g. indications of increased commitment to mental health care scale-up, through preparation of implementation plans, committing more resources and efforts to support reduction of stigma, discrimination and abuse). Details of the evaluations are presented in Additional file 5. Information about outcome indicators was generally not collected but when presented, was not robustly evaluated. For example, the outcome indicator “evidence of heightened awareness about the salience of improving mental health services and/or reduction in stigmatization” following mhLAP varied from “efforts to improve” one mental health centre in Liberia and “rehabilitation of homeless mentally ill citizens in some states” in Nigeria to “plans to review the mental health legislation are on-going” in Gambia [35]. More general ‘lessons learned’ were more frequently reported, which focused on the importance of building sustainable, high quality relationships and the need for continued mentoring and joint activities to support mental health systems strengthening.

#### Quality assessment

Quality assessment of quantitative aspects using the EPHPP tool found all included studies to be ‘weak,’ with between 3 and 6 ‘weak’ ratings across 8 domains. Assessment of qualitative aspects found 12 included papers to be of low quality (scoring less than 7 out of 12), and two more recent papers to be of medium quality (scoring between 7 and 9 out of 12: [30, 31]).

### Excluded papers

Many papers were excluded because they referred to service level capacity-building, for example, building capacity in health workers to deliver mental health care. In several excluded papers, policy-making and planning activities to strengthen mental health systems were described, for example, raising awareness and advocating for change, establishing a Ministry of Health mental health co-ordination desk or implementing guidelines for reliable drug supplies, but no specific interventions to build the capacity of policy-makers and planners to implement these activities were described [39–43]. Similarly, four of the 28 papers identified outside the database search described innovative mental health development work in Latin America and the Caribbean [44–47]. Despite detailed documentation of the work’s historical context, political shifts and clinical detail, the lack of description of capacity-building of policy-makers and planners prohibited their inclusion.

In some cases, an outside agency carried out the system strengthening activity [40, 41] and, in other cases, a ‘sustained policy dialogue’ was undertaken [39, 43]. These studies were excluded because the policy dialogues were not described or evaluated. Similarly, WHO developed training materials (including PowerPoint presentations, workshop facilitation guidelines and case materials) to build capacity for mental health policy and service development for LMICs, linked to the 13 modules of its Mental Health Policy and Service Guidance Package [7]. These resources provide practical, step-by-step guidance for developing national mental health policies, information systems and strengthening human resources and can be adapted for training purposes. However, since no formal evaluation of their impact on policy-makers and planners was published, these interventions were not included in this review.

## Discussion

This systematic review was prompted by the need for evidence on the most effective approaches to building capacity of mental health policy-makers and planners in LMICs. Health policy and systems research methods as they pertain to LMICs have been a neglected field [48]. Challenges identified include under-funding, fear that health policy and systems research does not yield generalizable findings, poor quality and a lack of alignment between research foci and policy-makers’ needs. The negative impact of a weak evidence base in health systems research on clinical outcomes, such as those specified in the Millennium Development Goals, is not a new observation [49].

Our study demonstrates that a restricted number of capacity-building interventions for policy-makers and planners in LMICs to strengthen mental health systems have been developed, implemented, evaluated and described in published literature. However, the quality of evidence evaluating the effectiveness of the different capacity-building approaches trialled is generally low and little attempt has been made to isolate the impact of the intervention, distinct from other contextual factors. Nevertheless, several features of effective capacity-building (Table 2) and evaluation of such interventions (Table 3) are suggested by this review.

**Table 2:** Features of effective capacity-building suggested by this review

• Meaningful partnership, in some cases between better and less well-resourced settings. • Establishing sustainable long-term support networks both between regions of mental health care delivery within a country and between nations, which develop over periods of years. • Technical and advisory support materials founded on regional and national experience and expertise, to assist with local mental health policy-making and planning. • Local statutory co-operation with interventions, with opportunities to expand successful models. • Emphasis on training, education and empowerment of leadership, including through formal qualifications. • Multidisciplinary investment targeting a range of mental health professionals and stakeholders, including service users, carers and staff in allied sectors such as social services.

**Table 3:** Recommendations for evaluation of capacity-building interventions suggested by this review

• It would be preferable to implement and evaluate specific interventions in isolation wherever possible, in order to ensure that the impact of individual components of more multifaceted programs can be assessed. • The use of a systematic evaluation framework incorporating defined process, output and outcome indicators and external reviewers where possible, is recommended. • It is recommended that intervention programs devise instruments to assess the mental health system strengthening competencies of stakeholders before and after participation. • Identification of the optimum duration of training for the target group of mental health policy-makers and planners in different LMIC settings is crucial, given the tension between delivering adequate content and feasible roll-out to busy professionals. • The impact of staff turnover in course leadership positions on the outcome of capacity-building interventions requires further investigation. • In-depth qualitative evaluation, including use of formal case study methodology, interviews and focus groups with staff who did and did not participate in the intervention, service users, carers and community members may provide valuable insights into mechanisms of impact, but may be more locally relevant. • Recent proposals that capacity-building interventions should be assessed according to their effect on access to, interaction with and receptivity to research evidence, at the level of the individual, the organization and the institution should be explored with specific reference to mental health policy-makers and planners.	

Management and leadership skills for general health systems strengthening, targeting decision-makers in low-income countries, are one area which has begun to be explored, with evidence of effective programs in post-conflict Liberia [50]. The importance of tailoring leadership capacity-building interventions to the stakeholder context was identified by a qualitative study involving health care leaders from Ethiopia, Ghana, Liberia and Rwanda [51]. The authors criticized the largely Western-centric literature on leadership capacity-building for neglecting the historical, political, and socio-cultural aspects of leadership in different countries, with health care leadership in sub-Saharan Africa still less well-studied. It is, perhaps, unsurprising, that capacity-building for mental health leadership in LMICs has been even less thoroughly researched.

This review identified two courses building mental health leadership capacity for participants from nine African [35] and 18 Asian countries [32–34]. Process and output indicators supported the favorability and perceived relevance of both leadership courses and reported that delegates went on to engage in mental health strengthening activities in their countries of origin.

Disease-specific health investment can distort national priorities and allocation of staff [15]. As a result, the need for renewed focus on wider health systems strengthening has been increasingly supported [14]. In each capacity-building intervention identified by this review, specific training for policy-makers and planners was embedded within a larger program of activities to support mental health system strengthening. A range of inter-related achievements were reported by each multi-faceted intervention, including staff attitude change [23, 31], increased funding [24, 25] policy change [26–29], education and qualifications for stakeholders [30], pursuit of doctoral and other research by participants [32–34] and creation of national [35] and international stakeholder councils or consortia [36]. These findings support a ‘systems thinking’ approach, whereby unified interventions collaboratively target health care, education, research and policy-making, acknowledging the dynamic nature of health services as complex adaptive systems [13]. The need for longer-term mentoring and partnership, often between low and high income countries [23, 25, 30, 32–35], underpinning shorter-term interventions, built on high quality relationships, emerged as being particularly important for success. Many of the partnerships reported developed over years, usually for time periods dictated by the duration of external funding. While the optimal duration of partnership could not be inferred by this review, the importance of long-term partnership working was evident across the range of published studies.

However, despite yielding demonstrable benefits, the complex systems-wide approach to capacity-building reported by each study rendered isolated evaluation of specific components difficult, especially given uncertainty about which ‘active ingredients’ of the intervention contribute to outcomes. The International Mental Health Leadership Programme (iMHLAP) model prioritized leadership skills, but also provided training in a broad range of topics relevant to mental health system strengthening [32, 33, 35]. Training courses developed for provincial and district-level planners in Kenya and Tanzania [26–29] were not well-described, but focused on practical skills in mental health service planning, budgeting and co-ordination. The difficulty associated with presenting evidence for an individual capacity-building intervention for mental health policy-makers and planners epitomizes the neglect of health policy and systems research methods in LMICs [48].

No study presented evaluation findings on the impact of specific capacity-building initiatives upon the mental health system strengthening competencies of participants. Furthermore, the optimal duration of training workshops for this target group remains unclear, having ranged from several days in Kenya and Tanzania to two weeks in West Africa and four weeks in Asia; a balance is required between adequate content and duration and feasible roll-out to busy professionals. Neither was evidence presented for the impact of course accessibility and staff turnover in course leadership positions on the outcomes of such interventions. As has been previously argued, the weakness of health policy and systems research methods in LMICs may compromise the efficacy and longer-term impact of capacity-building programs [49].

The evaluation of capacity-building interventions embedded in larger programatic interventions is inevitably complex. The indicators identified by this review provide potential starting points for more in-depth evaluation. The use of a systematic evaluation framework, as used in the capacity-building intervention targeting substance misuse in Russia, may be less susceptible to bias than more descriptive accounts of program outcomes [23]. Qualitative exploration, incorporating semi-structured interviews with all individuals responsible for policy-making and planning of mental health services, including those who did not participate in specific interventions, may yield insights into the mechanisms by which capacity-building activities work [10].

Training evaluation offers an important process indicator that may help to isolate the immediate impact of capacity-building interventions for mental health policy-makers and planners. Formal case study methodology may also facilitate a systematic, in-depth but contextualised evaluation of impact [3–52]. A recent study of capacity-building activities to apply evidence from general health research to policy-making in four LMICs proposed a framework for measurement and evaluation [8]. Advocating a more systematic approach to evaluation, the authors propose that capacity-building interventions should be assessed in terms of the extent that they increase access to, interaction with and receptivity to research evidence, with respect to the three levels of the individual, the organization and the institution. For example, increased access to research papers works at the individual level, whereas evidence-based policies work at the institutional level. A similar framework could provide a more systematic approach to evaluating interventions specifically focused on capacity-building of mental health policy-makers and planners.

A potential limitation of our review could be the tendency for reports on capacity-building for this target group of policy-makers and planners to be located within the grey literature, so that we may have missed relevant studies.

## Conclusions

Despite international consensus regarding the need for in-depth, system-wide capacity-building interventions for mental health policy-makers and service planners in LMICs, there is a paucity of published research studies and service evaluations in this field. This systematic review identified just fourteen studies describing nine different approaches, focused largely on systems-wide interventions to enhance mental health services, or on leadership courses. All studies shared a broad focus on partnership and high quality relationships, in keeping with the shift in focus from disease-specific investment to wider health system strengthening to build mental health capacity in LMICs. The preliminary evaluations presented here support a positive impact on process and output indicators. However, systematic research and evaluation methods were absent from all studies, compromising the potential to draw conclusions regarding outcomes on mental health system strengthening. This review demonstrates the need for high-quality capacity-building interventions for mental health policy-makers and planners, assessed using rigorous mixed methods to capture both quantitative and qualitative indices, as part of longitudinal evaluation of multi-faceted partnership approaches to mental health system strengthening in LMICs.

## Additional files

Additional file 1: Search strategy. Detail of search terms used and databases searched (DOCX 15 kb)
Additional file 2: Data extraction form. Table showing domains of data extracted during review of papers (DOCX 12 kb)
Additional file 3: Quality Assessment of Quantitative Studies. Table summarizing systematic evaluation of quantitative studies included in the review (DOCX 25 kb)
Additional file 4: Quality Assessment of Qualitative studies. Table summarizing systematic evaluation of qualitative studies included in the review (DOCX 35 kb)
Additional file 5: Summary of systematic review findings. Table of data extracted from articles included in the review (DOCX 28 kb)

## Abbreviations

CDC: Centers for Disease Control and Prevention
CNCD-Africa: Centre for Non-Communicable Disease, Africa
EPHPP: Effective public health practice project
iMHLAP: International Mental Health Leadership Progam
ISC: Inter-sectoral Steering Committee
LMICs: Low- and middle-income countries
mhLAP: Mental health leadership and advocacy
MNS: Mental, neurological and substance abuse
NCD: Non-communicable disease
NGO: Non-government organization
NSC: National Stakeholders Council
WHO: World Health Organization
