# Supplementary material for: Building the capacity of policy-makers and planners to strengthen mental health systems in low- and middle-income countries: a systematic review
Source: BMC Health Serv Res. 2016 Oct 21;16:601. doi: 10.1186/s12913-016-1853-0 (PMC5073499; doi:10.1186/s12913-016-1853-0)
Supplement: Additional file 1: — Search strategy. Detail of search terms used and databases searched (DOCX 15 kb) [file 12913_2016_1853_MOESM1_ESM.docx]

Additional file 1: Search strategy

| **(1) Search terms**  **“Capacity-building” AND “health systems and services” AND “mental health” AND “policy-makers, service planners and managers” AND “LMICS”**  **Capacity-building**  #1 Search: (exp Capacity Building/ OR exp Education/ OR exp Inservice training/) OR (capacity-building OR build capacity OR building capacity OR education OR training OR capacity development OR developing capacity OR development of capacity OR teaching).mp.  **Health systems and services**  #2 Search: (exp Delivery of Health Care/ OR exp Health Policy/ OR exp Health Services/ OR exp Mental Health Services/ OR exp Community Mental Health Services/) OR (delivery of health care OR health care delivery OR health system strengthening OR health policy OR health policies OR health system OR health systems OR health services OR mental health system OR mental health systems OR mental health services OR community mental health services).mp.  **Mental health**  #3 Search: (exp Mental health/ OR exp Mental Disorders/) OR (mental health OR mental disorder OR mental disorders OR mental illness OR mental illnesses).mp.  **Policy-makers, service planners and managers**  #4 Search: (exp Policy Making/ OR exp Health Planning/ OR exp Decision Making) OR (policy makers OR service planners OR service managers OR health managers OR health facility managers OR health planners OR health facility planners OR decision makers).mp.  **LMICs**  #5 Search:  (developing OR less developed OR under developed OR underdeveloped OR middle income OR low income OR lower income).mp. AND (countr* OR nation* OR population* or world).mp.  OR  (transitional OR developing OR less developed OR lesser developed OR under developed OR underdeveloped OR middle income OR low income OR lower income).mp. AND (economy OR economies).mp.)  OR  ((low*).mp. AND (gdp OR gnp OR gross domestic OR gross national).mp.) OR (lmic OR lmics OR lamics OR lamic OR third world OR lami countries OR lami country).mp. OR (transitional country OR transitional countries).mp.  OR  Exp Developing Countries/  OR  (Afghanistan or Albania or Algeria or Angola or Antigua or Barbuda or Argentina or Armenia or Armenian or Aruba or Azerbaijan or Bangladesh or Benin or Byelarus or Byelorussian or Belarus or Belorussian or Belorussia or Belize or Bhutan or Bolivia or Bosnia or Herzegovina or Hercegovina or Botswana or Brazil or Bulgaria or Burkina Faso or Burkina Fasso or Upper Volta or Burundi or Urundi or Cambodia or Khmer Republic or Kampuchea or Cameroon or Cameroons or Cameron or Camerons or Cape Verde or Central African Republic or Chad or Chile or China or Colombia or Comoros or Comoro Islands or Comores or Mayotte or Congo or Zaire or Costa Rica or Cote d Ivoire or Ivory Coast or Croatia or Cuba or Cyprus or Czechoslovakia or Czech Republic or Slovakia or Slovak Republic or Djibouti or French Somaliland or Dominica or Dominican Republic or East Timor or East Timur or Timor Leste or Ecuador or Egypt or El Salvador or Eritrea or Estonia or Ethiopia or Fiji or Gabon or Gabonese Republic or Gambia or Gaza or Georgia Republic or Georgian Republic or Ghana or Gold Coast or Grenada or Guatemala or Guinea or Guam or Guiana or Guyana or Haiti or Honduras or India or Maldives or Indonesia or Iran or Iraq or Jamaica or Jordan or Kazakhstan or Kazakh or Kenya or Kiribati or Korea or Kosovo or Kyrgyzstan or Kirghizia or Kyrgyz or Kirghiz or Kirgizstan or Lao PDR or Laos or Latvia or Lebanon or Lesotho or Basutoland or Liberia or Libya or Lithuania or Macedonia or Madagasca or Malagasy or Malaysia or Malaya or Malay or Sabah or Sarawak or Malawi or Nyasaland or Mali or Marshall Islands or Mauritania or Mauritius or Agalega Islands or Mexico or Micronesia or Middle East or Moldova or Moldovia or Moldovian or Mongolia or Montenegro or Morocco or Ifni or Mozambique or Myanmar or Myanma or Burma or Namibia or Nepal or Netherlands Antilles or New Caledonia or Nicaragua or Niger or Nigeria or Mariana Islands or Oman or Muscat or Pakistan or Palau or Palestine or Panama or Paraguay or Peru or Philippines or Philipines or Phillipines or Phillippines or Romania or Rumania or Roumania or Russia or Russian or Rwanda or Ruanda or Saint Kitts or St Kitts or Nevis or Saint Lucia or St Lucia or Saint Vincent or St Vincent or Grenadines or Samoa or Samoan Islands or Navigator Island or Navigator Islands or Sao Tome or Senegal or Serbia or Montenegro or Seychelles or Sierra Leone or Slovenia or South Africa or Sri Lanka or Ceylon or Solomon Islands or Somalia or Somaliland or South Africa or Sudan or Suriname or Surinam or Swaziland or Syria or Tajikistan or Tadzhikistan or Tadjikistan or Tadzhik or Tanzania or Thailand or Togo or Togolese or Tonga or Trinidad or Tobago or Tunisia or Turkey or Turkmenistan or Turkmen or Uganda or Ukraine or Uruguay or USSR or Soviet Union or Union of Soviet Socialist Republics or Uzbekistan or Uzbek or Vanuatu or New Hebrides or Venezuela or Vietnam or Viet Nam or West Bank or Yemen or Yugoslavia or Zambia or Zimbabwe or Rhodesia).mp.  **(2) Databases included**  Medline, Embase, PsycInfo, Web of Knowledge, LILACS, Eldis, Scileo and Scopus. |
| --- |
