# Supplementary material for: Building the capacity of policy-makers and planners to strengthen mental health systems in low- and middle-income countries: a systematic review
Source: BMC Health Serv Res. 2016 Oct 21;16:601. doi: 10.1186/s12913-016-1853-0 (PMC5073499; doi:10.1186/s12913-016-1853-0)
Supplement: Additional file 2: — Data extraction form. Table showing domains of data extracted during review of papers (DOCX 12 kb) [file 12913_2016_1853_MOESM2_ESM.docx]

# Additional file 2: Data Extraction Form

| Authors | Any author from LMIC? | Year of publication | Type of publication | Countries involved | Study design | Capacity building participant group | Sample size | Type, subject and approach/ framework (if any) of capacity building | Type of evaluation of involvement (if any) | Type of data collected | Summary of findings | Comments on quality^1^ |
| --- | --- | --- | --- | --- | --- | --- | --- | --- | --- | --- | --- | --- |
|  |  |  |  |  |  |  |  |  |  |  |  |  |
|  |  |  |  |  |  |  |  |  |  |  |  |  |
|  |  |  |  |  |  |  |  |  |  |  |  |  |
|  |  |  |  |  |  |  |  |  |  |  |  |  |
|  |  |  |  |  |  |  |  |  |  |  |  |  |
|  |  |  |  |  |  |  |  |  |  |  |  |  |
|  |  |  |  |  |  |  |  |  |  |  |  |  |

1 Include, for example, whether the aims are clearly stated; whether the design is appropriate to the aims; evidence of reliability/validity of measures used; and whether the selection of the sample is relatively unbiased.
