# Supplementary material for: Building the capacity of policy-makers and planners to strengthen mental health systems in low- and middle-income countries: a systematic review
Source: BMC Health Serv Res. 2016 Oct 21;16:601. doi: 10.1186/s12913-016-1853-0 (PMC5073499; doi:10.1186/s12913-016-1853-0)
Supplement: Additional file 3: — Quality Assessment of Quantitative Studies. Table summarizing systematic evaluation of quantitative studies included in the review (DOCX 25 kb) [file 12913_2016_1853_MOESM3_ESM.docx]

Additional file 3: Quality Assessment of Quantitative Studies
