# Supplementary material for: Building the capacity of policy-makers and planners to strengthen mental health systems in low- and middle-income countries: a systematic review
Source: BMC Health Serv Res. 2016 Oct 21;16:601. doi: 10.1186/s12913-016-1853-0 (PMC5073499; doi:10.1186/s12913-016-1853-0)
Supplement: Additional file 4: — Quality Assessment of Qualitative studies. Table summarizing systematic evaluation of qualitative studies included in the review (DOCX 35 kb) [file 12913_2016_1853_MOESM4_ESM.docx]

Additional file 4: Quality Assessment of Qualitative studies

Twelve review criteria were used to assess the quality of qualitative studies (Harden et al.; 2009): 1. Were the aims and objectives clearly reported? 2. Was there an adequate description of the context in which the research was carried out? 3. Was there an adequate description of the sample and the methods by which the sample was identified and recruited? 4. Was there an adequate description of the methods used to collect data? 5. Was there an adequate description of the methods used to analyse data? 6. Were there attempts to establish the reliability of the data collection tools (for example, by use of interview topic guides)? 7. Were there attempts to establish the validity of the data collection tools (for example, with pilot interviews)? 8. Were there attempts to establish the reliability of the data analysis methods (for example, by use of independent coders)? 9. Were there attempts to establish the validity of data analysis methods (for example, by searching for negative cases)? 10. Did the study use appropriate data collection methods for helping people to express their views? 11. Did the study use appropriate methods for ensuring the data analysis was grounded in the views of people? 12. Did the study actively involve relevant groups in its design and conduct?
