# Supplementary material for: Building the capacity of policy-makers and planners to strengthen mental health systems in low- and middle-income countries: a systematic review
Source: BMC Health Serv Res. 2016 Oct 21;16:601. doi: 10.1186/s12913-016-1853-0 (PMC5073499; doi:10.1186/s12913-016-1853-0)
Supplement: Additional file 5: — Summary of systematic review findings. Table of data extracted from articles included in the review (DOCX 28 kb) [file 12913_2016_1853_MOESM5_ESM.docx]

Additional file 5: Summary of Systematic review findings

| Reference | Capacity-building intervention | Countries involved | Capacity-building participant group | Sample  size | Capacity-building evaluation  measures | Achievements and Lessons Learned |
| --- | --- | --- | --- | --- | --- | --- |
| [22] | Mental health system strengthening. | Russia | Personnel with responsibilities for policy-making and implementation, including senior clinical nursing and medical staff. | Not specified. | External review assessed documentary analysis of subsequent funding applications, project manager reports, telephone and face-to-face interviews with UK partners and Russian participants.  Collaborative ‘insider’ evaluation, from interviews with Russian participants and policy-makers, clinical observations and literature review. | Achievements:  The evaluation acknowledged that direct impact on patient outcomes could not be quantified, but identified changes which followed the project, to which they could in part be attributed, as follows:   1. Increased awareness of and support for changes to drug abuse services, resulting in a St Petersburg strategy on substance use disorders and enhanced funding by the local Health Committee. 2. Psycho-social approaches and task sharing between doctors and nurses became commonplace in substance misuse treatment facilities, including those which had not participated directly. 3. Greater interest in and uptake of specialist training for nurses, fostering improved multidisciplinary team working. 4. Higher-level institutional changes took place to reinforce the importance of multidisciplinary care.   Lessons learned:   - On-going staff turnover among policy-makers and political and economic changes affect the stability and scope of such interventions, long-term. |
| [23] | Mental health (substance misuse) system strengthening: project ORCHID | India | Community groups, service providers, NGOs, state and national AIDS agencies. | Not specified. | Descriptive quantitative results from service-user engagement. | Achievements:   1. Project ORCHID developed guidelines, training manuals, monitoring resources and procedures which have been integrated into national government strategy. 2. It was integrated into other health promotion initiatives pertaining to HIV and other infectious diseases. 3. There is an ongoing process of funding transition to government sources, with 25% of programs now state-funded.   Lessons learned:   - Service users contributed valuable insights into more flexible and creative service planning to scale-up harm reduction for injecting opioid use. - Community leadership offered an innovative perspective on the best service management model and ensured sustainability of the care delivered. - Data collection, field monitoring and clearly-defined roles are vital to effective service delivery. - Structured advocacy via the community and influential persons is the best way to manage barriers to service delivery. - Smaller-scale local projects should share their findings with the government for the purpose of scaling-up further. |
| [24] | Mental health system strengthening. | Russia | Staff of 8 non-government organizations (NGOs). | Not specified. | Interviews, focus groups, observations in clinical, team and inter-sector settings. | Achievements:   1. Seven NGOs created self-help groups and all eight established programs for engaging people with mental health problems in meaningful activity or employment. 2. NGOs bid for and received external funding for eight new housing and job services in the region. 3. Two new NGOs were created during the project’s duration. 4. Inter-sectoral Steering Committees (ISCs) began to involve service users and NGOs in service planning and delivery   Lessons learned   - Local and national policy can be influenced through demonstration, via successful pilot projects. - Changing funder priorities limited the potential for in-depth outcomes analysis. - Politically-driven long-term funding regulation reforms are required in some countries to facilitate improvements in mental health care. For example de-institutionalization in Russia is limited by restrictions on transferring budgets from health to social care departments. |
| [25-27] | Mental health system strengthening. | Kenya | Psychiatrists, district psychiatric nurses and district public health nurses. | 4 courses were delivered to 8 psychiatrists and 200 district psychiatric and district public health nurses. | Descriptive account only. | Achievements:   1. Mental health was incorporated into local annual operational plans, which feed into the national annual operational plan. 2. Secondary care psychiatric and public health nurses engaged in supervision and coordination of primary mental health care workers. 3. In-depth situation analysis and needs assessment took place. 4. Mental health policy guidelines were created. 5. Mental health was incorporated into plans for health sector reform and the national Package of Essential Health Interventions. 6. A set of roles and responsibilities for different mental health workers was agreed. 7. Work was undertaken to ensure medication access for primary care, including creation of a National Essential Drugs List. 8. Partnerships were developed with other government ministries, including police, prisons, schools and social services. 9. Public mental health awareness-raising activities were undertaken.   Lessons learned:   - A comprehensive program of inter-connected and planned projects at a range of levels from local to national and across sectors is key to successful implementation. - On-going in-depth policy dialogue is crucial throughout such projects, with long-term involvement of external agencies especially important. - Sustainability must be planned for from the outset. The program should support the development of local services from government budgets rather than creating services with fixed-term funding which is unlikely to continue. - Frequent ministerial staff turnover, changes in government departmental organization and economic and political instability are challenges to successful implementation. |
| [28] | Mental health system strengthening. | Tanzania | District mental health coordinators. | Not specified | Descriptive account only. | Achievements:   1. Mental health funding was enhanced, following training of district mental health coordinators in integrating budgets into district health plans. 2. In-depth situation analysis took place, constructing a mental health profile of Tanzania. 3. The Section of Mental Health was established in the Directorate of Curative Services and the National Mental Health Resource Centre of Tanzania was founded. 4. Mental health was incorporated into the health sector reform plan. 5. Mental health policy guidelines were placed in the National Package of Essential Health Interventions, the Medium-Term Expenditure Framework and the National Strategy for Non-Communicable Disease. 6. A National Essential Drugs List highlighting key psychotropic medications was compiled. 7. Mental health training was delivered to primary care workers, with follow-up supervision and monitoring by local mental health coordinators, who were themselves trained. 8. Focus groups with local leaders enabled improved public awareness of mental health problems and increased support for mental health care interventions. |
| [29] | Training and education of key stakeholders | Nicaragua, Chile | Professionals working in primary care, mental health care and addictions, interested academics and NGO staff. | 47 professionals contributed to the needs assessment. Workshops were attended by 79 individuals. | Descriptive account only | Achievements:   1. A needs assessment identified stakeholder priorities and learning needs pertaining to inter-professional teamwork and overcoming mental health stigma. 2. The partnership strengthened collaboration between Nicaragua and Canada. 3. Participants rated workshops highly for satisfaction and learning. 4. A diploma aimed at service providers, planners and managers was implemented and a Masters program launched.   Lessons learned:   - The program’s impact was limited by insufficient mental health and research funding. - The distance between Nicaragua and Canada prevented more consistent collaboration. - There was a lack of translation at the government level of a commitment to integrate mental health care into primary care, into action. |
| [30] | Children’s mental health system strengthening | Russia | Medical, psychological, and educational service providers supported by the Ministries of Social Policy and Health. | 8 pilot sites and 5 participant sites (not selected to be pilot sites but able to send staff to participate in training), with 75 regular participants and over 300 staff attending open lectures and seminars. | Descriptive account only. | Achievements:   1. Specialists attending training regularly showed improved understanding and use of early intervention principles. 2. Family support and early intervention visits increased over three years from 0 to almost 1000 per month. 3. Effects were seen at both pilot and participant sites. 4. Numbers of children without registered parent care, removal of children from their parents’ care and numbers of children living in orphanages reduced during the project.   Lessons Learned:   - The goal of longitudinal educational assessment was affected by lack of familiarity with standardized testing and anxiety about detrimental effects on school acceptance. This indicated a need for more work on the importance of documentation. - Ministerial data about children’s outcomes were incomplete, indicating a need for a more efficient regional system of data collection. This may have resulted from pressure on ministries to collect activity and resource-focused information rather than clinical outcomes data. |
| [31-33] | International Mental Health Leadership program (iMHLP). | 18 countries in Asia and the Pacific. | Mental health professionals, managers, planners and policy-makers. | Over 170 participants. | Description of iMHLP graduates’ activities and experiences of running the program. | Achievements:   1. A network of over 170 alumni across 18 countries in the Asia-Pacific region was established. 2. Numerous former participants now hold positions in health ministries, universities, the World Health Organization and others. 3. Several course alumni went on to pursue PhD-level studies and contributed to projects which research and develop mental health systems.   Lessons learned:   - The importance of building sustainable, long-term partnerships. - The need to work on the quality of the relationships underpinning partnerships. - The requirement for partnerships based on “honesty, mutual respect and trust, supplemented by a joint commitment to equity and to protecting the rights of people with mental illness”. |
| [34] | Mental Health Leadership and Advocacy Program (mhLAP). | Ghana, Nigeria, Liberia, Sierra Leone and The Gambia, with participants from a further four African countries. | Mental health leaders, government staff at middle and high levels, policy-makers, community leaders, service user and carer organizations. | 96 participants | Defined process, output and outcome indicators. | Achievements:   1. Creation of National Stakeholder Councils (NSC): multidisciplinary panels, incorporating non-government organizations, health workers and media, focused on local priorities for mental health advocacy.   Process indicators:   1. In each country, engagement meetings took place with policy-makers and planners, some of whom became members of the local NSC.   Output indicators:   1. In each country, NSC members participated in advocacy activities and meetings with government officials, including legislative reviews.   Outcome indicators:   1. A local situation analysis of mental health services was performed in each country. 2. Diminished stigmatization towards people with mental health problems and increased public understanding of the need for better mental health services were demonstrated.   Lessons learned :   - Policy-maker and government responses to mental health advocacy by NSCs were slower than hoped. - Working with the local World Health Organization (WHO) office in some countries ensured that mhLAP activities received associated support and recognition. - Gradual implementation of mhLAP in each country enabled initial lessons learned to inform the program in subsequent iterations. - Challenges in communication and logistics were identified across the range of participating countries. |
| [35] | CNCD-Africa | Nigeria, South Africa, Ghana, Canada, Uganda, Zambia, Congo and Kenya | Representatives of civil society organizations, professional associations and international institutions | Symposia on NCDs were attended by 115 participants. | Descriptive account only. | Achievements:   1. The consortium succeeded in convening high profile meetings, knowledge generation and sharing through production and promotion of policy documents, advocacy through World Health Organization and United Nations fora and networking electronically and through social media.   Lessons learned:   - Retaining the interest of network partners is essential. - High profile events and obtaining the support of local and international organizations interested in NCDs in sub-Saharan Africa are vital to retaining partners’ interest in the network. - Existing regional platforms are an effective means of publicizing successful interventions and strategies. - Partnerships and stakeholder participation are crucial for joint inception, planning, implementation and evaluation of interventions but require incentives. - Robust, locally-responsive, relevant regional global partnerships can yield innovative financing. |
